# Association of body mass index with mortality and functional outcome after acute ischemic stroke

**DOI:** 10.1038/s41598-017-02551-0

**Published:** 2017-05-31

**Authors:** Weiping Sun, Yining Huang, Ying Xian, Sainan Zhu, Zhirong Jia, Ran Liu, Fan Li, Jade W. Wei, Ji-Guang Wang, Ming Liu, Craig S. Anderson

**Affiliations:** 1 Department of Neurology, Peking University First Hospital, Beijing, China; 2 Department of Neurology, Duke Clinical Research Institute, Duke University Medical Center, Durham, NC USA; 3 Department of Biostatistics, Peking University First Hospital, Beijing, China; 4 The George Institute for Global Health, Royal Prince Alfred Hospital and the University of Sydney, Sydney, Australia; 5 The Center for Epidemiological Studies and Clinical Trials, Ruijin Hospital, Shanghai Jiao Tong University School of Medicine, Shanghai, China; 6 Department of Neurology, West China Hospital, Sichuan University, Chengdu, China

## Abstract

The relation between obesity and stroke outcome has been disputed. This study was aimed to determine the association of body mass index (BMI) with mortality and functional outcome in patients with acute ischemic stroke. Data were from a national, multi-centre, prospective, hospital-based register: the ChinaQUEST (Quality Evaluation of Stroke Care and Treatment) study. Of 4782 acute ischemic stroke patients, 282 were underweight (BMI < 18.5 kg/m^2^), 2306 were normal-weight (BMI 18.5 to < 24 kg/m^2^), 1677 were overweight (BMI 24 to <28 kg/m^2^) and 517 were obese (BMI ≥ 28 kg/m^2^). The risks of death at 12 months and death or high dependency at 3 and 12 months in overweight (HR: 0.97, 95% CI: 0.78–1.20; OR: 0.93, 95% CI: 0.80–1.09; OR: 0.95, 95% CI: 0.81–1.12) and obese patients (HR: 1.07, 95% CI: 0.78–1.48; OR: 0.96, 95% CI: 0.75–1.22; OR: 1.06, 95% CI: 0.83–1.35) did not differ from normal-weight patients significantly after adjusting for baseline characteristics. Underweight patients had significantly increased risks of these three outcomes. In ischemic stroke patients, being overweight or obese was not associated with decreased mortality or better functional recovery but being underweight predicted unfavourable outcomes.

## Introduction

Obesity is an established risk factor for cardiovascular disease and poses a serious public health burden worldwide^1–4^. While epidemiological investigations have concluded that obesity is strongly associated with an increased risk of stroke in the general population^5, 6^, controversy exists regarding the relationship between obesity and outcome in patients who have had an established stroke^7–18^. Several articles report that increased body mass index (BMI) independently predicts a lower mortality after stroke or transient ischemic attack (TIA)^9–15^, but others have found no survival advantage in being overweight or obese after stroke^16–18^. Although Doehner *et al*. have found that obese patients have a better functional outcome than normal-weight patients following acute stroke^15^, the relation between BMI and functional disability is not well established. Such controversy raises doubts about the appropriateness of recommending weight loss for the secondary prevention of stroke in overweight and obese patients^16, 19^. Moreover, most investigations have been restricted to Western countries^8–16^, raising further uncertainty about the importance of BMI in Asian stroke patients who have relatively low BMI but high stroke incidence than in other populations^6, 20^.

The aim of our study was to investigate the association between BMI and stroke outcome, including mortality and functional measures, in a broad range of hospitalized patients with ischemic stroke in China.

## Methods

### Patients and study protocol

This study was on data from the ChinaQUEST (Quality Evaluation of Stroke Care and Treatment), a multicentre, prospective, hospital-registry (*n* = 62) study in 37 cities in China. The design and conduct of the ChinaQUEST has been described previously^21, 22^. In brief, consecutive patients (aged ≥ 15 years) admitted to a hospital within 30 days from onset of acute stroke due to ischemic stroke or intracerebral hemorrhage were included during a 5-month period in 2006^22^. Patients with subarachnoid hemorrhage were excluded.

Patients were assessed 4 times during the course of the study, at admission, discharge, and approximately 3 and 12 months post-stroke^22^. Baseline information such as socio-demographic variables, medical history and clinical features of the index stroke, was obtained predominantly by face-to-face interviews. In-hospital details on diagnostic and treatment strategies were obtained through medical records (46%) and interviews with patients or their proxies (54%). Follow-up information about functional outcome as measured by the modified Rankin scale (mRS) was collected mainly by telephone interview (>80%) at 3 and 12 months. Death and cause of death were ascertained by family members (64.7%), a health professional (18.4%), records (e.g. medical, police [11.8%]), death certificate (3.5%) or other sources (1.5%).

Written informed consent was obtained from all patients or an appropriate family member (if the patient was unable to give it) to participate. The study was approved by the ethics committees of Peking University First Hospital (Beijing), Ruijin Hospital (Shanghai), Prince of Wales Hospital (Hong Kong), and The University of Sydney. Good Clinical Practice guidelines in accordance with the Declaration of Helsinki were used and the privacy of patients was strictly protected.

### Variables of interest and outcomes

BMI was calculated from height and weight (kilograms per square metre) measured by nurses or obtained from the patient or the accompanying relatives on admission. The patient population was sub-grouped into the following categories according to the Chinese Guidelines on Prevention and Control of Obesity in Adults^23, 24^: underweight (BMI < 18.5 kg/m^2^), normal weight (BMI 18.5 to <24 kg/m^2^), overweight (BMI 24 to <28 kg/m^2^) and obese (BMI ≥ 28 kg/m^2^).

The outcomes included all-cause mortality at 12 months, and a composite of death or high dependency at 3 and 12 months post-stroke. High dependency was defined as a mRS score of 3–5.

Covariates considered included socio-demographic variables, medical history and clinical features of the index stroke, including: age, sex, partnership (living alone or living with a partner), education level (‘low level’, being illiterate or having only primary education; ‘high level’, having secondary education or higher) and annual household income (<20000 Chinese Yuan [CNY], approximately $2857 US; ≥20000 CNY; or declined to respond/unknown); medical history of hypertension, diabetes, hyperlipidaemia, atrial fibrillation, prior stroke/TIA, coronary artery disease (any history of heart attack and/or myocardial infarction, angina or coronary heart disease) either self-reported or diagnosed in hospital post-stroke; prior dependency, current cigarette smoking, and regular alcohol consumption within the 3 months before stroke onset; time of presentation at the hospital (<6 hour; ≥6 hour; or unknown), Glasgow coma scale (GCS) score on admission (‘mild’ 13–15; ‘moderate’ 8–12; or ‘severe’ 3–7) and Oxfordshire Community Stroke Project (OCSP) classification (total anterior circulation infarct [TACI]; partial anterior circulation infarct [PACI]; lacunar infarct [LACI]; posterior circulation infarct [POCI]; or unknown).

### Statistical analysis

Baseline characteristics were compared among different BMI subgroups using the one-way analysis of variance (ANOVA) and Chi-square test for continuous and for categorical variables as appropriate. The Cox proportional hazard model was performed to assess the association between BMI and all-cause mortality at 12 months. Similarly, logistic regression models were performed to evaluate the association between BMI and the composite outcome of death or high dependency at 3 and 12 months post-stroke. The association of BMI and high dependency was also evaluated in the survivors at 3 and 12 months after stroke. The distribution of causes of death in those who had died during the follow-up period was compared with Chi-square test among the BMI subgroups. We performed four additional analyses to assess the robustness of our main findings. First, we performed a sensitivity analysis using the World Health Organization (WHO) BMI classification criteria^25^, which defined underweight (BMI < 18.5 kg/m^2^), normal weight (BMI 18.5 to <25 kg/m^2^), overweight (BMI 25 to <30 kg/m^2^) and obese (BMI ≥ 30 kg/m^2^). Second, we restricted the study population to those who had not received thrombolysis treatment. Third, another definition of high dependency as a mRS score of 4–5 was used to validate the association between BMI and functional outcome^15^. Fourth, we performed a subgroup analysis according to smoking status (current smoker and current non-smoker). All *P* values were two-sided, with *P* < 0.05 considered statistically significant. All statistical analyses were performed using SPSS version 13.0 (SPSS Inc., Chicago, IL, USA).

## Results

### Baseline characteristics

There were 6508 acute stroke patients enrolled in the ChinaQUEST study. After excluding 1572 patients with intracerebral hemorrhage and 154 with uncertain diagnosis and missing data, a total of 4782 patients with acute ischemic stroke were included in our analysis. The mean age of those with ischemic stroke was 64.6 years (range 15–102 years) and 37.9% were female. Their average BMI was 23.8 ± 3.5 kg/m^2^. Of them, 282 patients (5.9%) were underweight, 2306 (48.2%) had normal weight, 1677 (35.1%) were overweight, and 517 (10.8%) were obese.

Baseline characteristics between the BMI subgroups are shown in Table 1. Underweight patients were older and more likely to have had little education, a low income, atrial fibrillation, prior dependency and low level of consciousness at baseline. Compared with normal-weight patients, obese patients were younger and more often had a medical history of hypertension, diabetes, hyperlipidaemia, previous stroke/TIA, and coronary artery disease. Overweight group had the highest proportions of male, current smoker and regular alcohol user.

**Table 1:** Baseline characteristics by body mass index subgroup.

	Underweight (n = 282)	Normal weight (n = 2306)	Overweight (n = 1677)	Obese (n = 517)	P Value
BMI (kg/m^2^)	17.2 ± 1.12	21.7 ± 1.45	25.7 ± 1.13	30.1 ± 2.57	<0.001
Age (years)	70.2 ± 12.8	65.4 ± 11.7	63.2 ± 11.4	63.2 ± 11.8	<0.001
Male Sex (%)	155 (55.0)	1455 (63.1)	1086 (64.8)	276 (53.4)	<0.001
Living alone (%)	20 (7.1)	107 (4.6)	75 (4.5)	32 (6.2)	0.124
Low education (%)	184 (65.2)	1121 (48.6)	702 (41.9)	219 (42.4)	<0.001
Annual household income (%)					0.001
<20,000 CNY	176 (62.4)	1371 (59.5)	909 (54.2)	268 (51.8)	
≥20,000 CNY	68 (24.1)	637 (27.6)	545 (32.5)	177 (34.2)	
Declined to respond/unknown	38 (13.5)	298 (12.9)	223 (13.3)	72 (13.9)	
Hypertension (%)	177 (62.8)	1635 (70.9)	1358 (81.0)	453 (87.6)	<0.001
Diabetes (%)	47 (16.7)	500 (21.7)	490 (29.2)	161 (31.1)	<0.001
Hyperlipidaemia (%)	89 (31.6)	936 (40.6)	838 (50.0)	305 (59.0)	<0.001
Prior stroke/TIA (%)	80 (28.4)	704 (30.5)	564 (33.6)	193 (37.3)	0.005
Coronary artery disease (%)	64 (22.7)	506 (21.9)	418 (24.9)	146 (28.2)	0.010
Atrial fibrillation (%)	42 (14.9)	216 (9.4)	142 (8.5)	42 (8.1)	0.005
Current smoker (%)	76 (27.0)	670 (29.1)	509 (30.4)	120 (23.2)	0.015
Regular alcohol use (%)	62 (22.0)	606 (26.3)	510 (30.4)	126 (24.4)	0.001
Prior dependency (%)	61 (21.6)	284 (12.3)	220 (13.1)	79 (15.3)	<0.001
Time from stroke onset to hospital presentation (%)					0.456
<6 hours	88 (31.2)	664 (28.8)	521 (31.1)	154 (29.8)	
≥6 hours	182 (64.5)	1516 (65.7)	1076 (64.2)	344 (66.5)	
Unknown	12 (4.3)	126 (5.5)	80 (4.8)	19 (3.7)	
Glasgow Coma Scale score on admission (%)					0.034
13–15	223 (81.4)	1957 (85.9)	1462 (88.0)	443 (87.7)	
9–12	32 (11.7)	216 (9.5)	132 (7.9)	35 (6.9)	
3–8	19 (6.9)	105 (4.6)	67 (4.0)	27 (5.3)	
OCSP classification on admission					0.544
TACI	32 (11.3)	244 (10.6)	176 (10.5)	54 (10.4)	
PACI	136 (48.2)	1138 (49.3)	853 (50.9)	250 (48.4)	
LACI	66 (23.4)	518 (22.5)	349 (20.8)	120 (23.2)	
POCI	30 (10.6)	302 (13.1)	239 (14.3)	69 (13.3)	
Unknown	18 (6.4)	104 (4.5)	60 (3.6)	24 (4.6)	

BMI, body mass index; CNY, Chinese Yuan; LACI, lacunar infarct; OCSP, Oxfordshire Community Stroke Project classification; PACI, partial anterior circulation infarct; POCI, posterior circulation infarct; TACI, total anterior circulation infarct; TIA, transient ischemic attack. 20000 CNY was equal to approximately $2857 US in 2006. Glasgow coma scale score on admission was available in 4718 patients (274 underweight patients, 2278 normal-weight patients, 1661 overweight patients and 505 obese patients).

### Body mass index and stroke outcomes

There were 484 patients who had died within 12 months after stroke. High dependency was found in 1221 patients at 3 months and in 942 patients at 12 months. Unadjusted analysis showed obese patients did not differ from patients of normal BMI significantly in the risks for death and death or high dependency after stroke. Overweight patients had a slightly lower proportion of death or high dependency at 12 months than normal-weight patients. Those who were underweight had the highest risks of the three endpoints (Table 2).

**Table 2:** Body mass index related to risk for death at 12 months, death or high dependency at 3 and 12 months.

	Underweight	Normal weight	Overweight	Obese
Death at 12 months				
No. of participants	282	2306	1677	517
No. of cases	58	233	146	47
HR (95% CI)				
Unadjusted	2.16 (1.62–2.88)	1.00	0.85 (0.69–1.05)	0.89 (0.65–1.22)
Adjusted	1.64 (1.22–2.22)	1.00	0.97 (0.78–1.20)	1.07 (0.78–1.48)
Death or high dependency at 3 months				
No. of participants	270	2262	1640	511
No. of cases	124	743	494	166
OR (95% CI)				
Unadjusted	1.74 (1.35–2.24)	1.00	0.88 (0.77–1.01)	0.98 (0.80–1.21)
Adjusted	1.46 (1.08–1.95)	1.00	0.93 (0.80–1.09)	0.96 (0.75–1.22)
Death or high dependency at 12 months				
No. of participants	272	2236	1626	504
No. of cases	124	691	454	157
OR (95% CI)				
Unadjusted	1.87 (1.45–2.41)	1.00	0.87 (0.75–1.00)	1.01 (0.82–1.25)
Adjusted	1.46 (1.08–1.96)	1.00	0.95 (0.81–1.12)	1.06 (0.83–1.35)

CI, confidence interval; HR, hazard ratio; OR, odds ratio. The adjusted analyses included the following covariates: age, sex, living partnership, education level, annual household income, medical history of hypertension, diabetes, hyperlipidaemia, atrial fibrillation, prior stroke or TIA, coronary artery disease, and prior dependency, current smoking status, regular alcohol consumption, time of presentation to hospital, Glasgow Coma Scale score on admission and Oxfordshire Community Stroke Project classification on admission.

After adjustment of all covariates, neither overweight (hazard ratio [HR]: 0.97, 95% confidence interval [CI]: 0.78–1.20; odds ratio [OR]: 0.93, 95% CI: 0.80–1.09; OR: 0.95, 95% CI: 0.81–1.12; compared with normal-weight group) nor obese (HR: 1.07, 95% CI: 0.78–1.48; OR: 0.96, 95% CI: 0.75–1.22; OR: 1.06, 95% CI: 0.83–1.35; compared with normal-weight group) was associated with death at 12 months, and death or high dependency at 3 and 12 months significantly. Underweight patients remained at significantly highest risk of poor outcome (Table 2).

In the surviving patients, there was no significant difference in the risk of high dependency in overweight and obese patients compared with normal-weight group, either (Table 3). In those who had died during the follow-up period, the distribution of causes of death did not differ significantly among the BMI subgroups (p = 0.200; Table 4).

**Table 3:** Body mass index related to risk for high dependency in surviving patients.

	Underweight	Normal weight	Overweight	Obese
High dependency at 3 months				
No. of participants	235	2114	1550	478
No. of cases	89	595	404	133
OR (95% CI)				
Unadjusted	1.56 (1.18–2.06)	1.00	0.90 (0.78–1.04)	0.98 (0.79–1.23)
Adjusted	1.34 (0.98–1.84)	1.00	0.92 (0.78–1.09)	0.94 (0.73–1.21)
High dependency at 12 months				
No. of participants	214	2003	1480	457
No. of cases	66	458	308	110
OR (95% CI)				
Unadjusted	1.50 (1.11–2.05)	1.00	0.89 (0.75–1.04)	1.07 (0.84–1.36)
Adjusted	1.19 (0.84–1.68)	1.00	0.93 (0.78–1.11)	1.07 (0.82–1.40)

CI, confidence interval; OR, odds ratio. The adjusted analyses included the following covariates: age, sex, living partnership, education level, annual household income, medical history of hypertension, diabetes, hyperlipidaemia, atrial fibrillation, prior stroke or TIA, coronary artery disease, and prior dependency, current smoking status, regular alcohol consumption, time of presentation to hospital, Glasgow Coma Scale score on admission and Oxfordshire Community Stroke Project classification on admission.

**Table 4:** Causes of death by body mass index subgroup.

	Underweight	Normal weight	Overweight	Obese	P Value
Cause of death					0.200
Cardiovascular	6 (10.3)	22 (9.4)	12 (8.2)	4 (8.5)	
Stroke	25 (43.1)	131 (56.2)	78 (53.4)	29 (61.7)	
Pulmonary embolism	0 (0)	1 (0.4)	4 (2.7)	0 (0)	
Infection	13 (22.4)	21 (9.0)	15 (10.3)	2 (4.3)	
Cancer	3 (5.2)	9 (3.9)	3 (2.1)	1 (2.1)	
Other causes	11 (19.0)	49 (21.0)	34 (23.3)	11 (23.4)	
Total	58	233	146	47	

P value by Chi-square test. Numbers in parentheses indicate percentages.

### Sensitivity analyses

The relation between BMI and the outcomes were consistent in sensitivity analyses (Table 5). The multivariable analyses showed the risks for death and death or high dependency in overweight and obese patients did not differ from normal-weight patients significantly when using the WHO BMI classification criteria, or excluding those patients who had received thrombolysis treatment. Using another definition of high dependency as a mRS score of 4–5 did not change the association between BMI and functional disability. The stratified analysis by smoking status showed no significant differences in these outcomes among normal-weight, overweight and obese patients in both subgroups.

**Table 5:** Sensitivity analysis: Body mass index and death at 12 months, death or high dependency at 3 and 12 months.

	Death at 12 months	Death or high dependency at 3 months	Death or high dependency at 12 months
	HR (95% CI)	OR (95% CI)	OR (95% CI)
Sensitivity analysis 1			
Underweight	1.63 (1.21–2.19)	1.46 (1.09–1.96)	1.46 (1.09–1.96)
Overweight	0.95 (0.76–1.18)	0.96 (0.82–1.13)	1.00 (0.85–1.18)
Obese	1.01 (0.62–1.66)	0.70 (0.48–1.01)	0.84 (0.58–1.22)
Sensitivity analysis 2			
Underweight	1.58 (1.16–2.15)	1.46 (1.08–1.97)	1.40 (1.03–1.90)
Overweight	0.96 (0.77–1.20)	0.93 (0.79–1.09)	0.95 (0.80–1.12)
Obese	0.97 (0.69–1.38)	0.93 (0.72–1.19)	1.05 (0.81–1.35)
Sensitivity analysis 3			
Underweight	1.64 (1.22–2.22)	1.46 (1.04–2.03)	1.88 (1.37–2.59)
Overweight	0.97 (0.78–1.20)	0.98 (0.81–1.18)	0.99 (0.82–1.20)
Obese	1.07 (0.78–1.48)	0.99 (0.74–1.32)	1.11 (0.83–1.47)
Sensitivity analysis 4			
Current smoker			
Underweight	2.01 (0.96–4.19)	1.37 (0.77–2.45)	0.98 (0.52–1.82)
Overweight	0.87 (0.51–1.49)	0.82 (0.60–1.12)	0.96 (0.70–1.33)
Obese	0.89 (0.34–2.36)	0.99 (0.59–1.68)	0.98 (0.56–1.72)
Current non-smoker			
Underweight	1.58 (1.13–2.20)	1.43 (1.01–2.02)	1.58 (1.12–2.24)
Overweight	0.97 (0.76–1.22)	0.96 (0.80–1.15)	0.94 (0.78–1.13)
Obese	1.11 (0.79–1.57)	0.94 (0.71–1.23)	1.07 (0.81–1.41)

CI, confidence interval; HR, hazard ratio; OR, odds ratio. The normal-weight group was the reference group. Sensitivity analysis 1 used the World Health Organization BMI criteria, which included 282 underweight patients (BMI < 18.5 kg/m^2^), 2889 normal-weight patients (BMI 18.5 to <25 kg/m^2^), 1408 overweight patients (BMI 25 to < 30 kg/m^2^) and 203 obese patients (BMI ≥ 30 kg/m^2^). Sensitivity analysis 2 excluded 234 patients who had received thrombolysis treatment, leaving 274 underweight patients, 2210 normal-weight patients, 1579 overweight patients and 485 obese patients in the analysis. Sensitivity analysis 3 redefined high dependency as a modified Rankin scale score of 4–5. Sensitivity analysis 4 was a stratified analysis according to smoking status (current smoker and current non-smoker). In 1375 patients of current smoker, there were 76 underweight patients, 670 normal-weight patients, 509 overweight patients and 120 obese patients. In 3407 patients of current non-smoker, there were 206 underweight patients, 1636 normal-weight patients, 1168 overweight patients and 397 obese patients.

## Discussion

The present study did not find any outcome advantage associated with being overweight or obese at the time of experiencing an acute ischemic stroke. The risks of death and functional disability in overweight and obese patients did not differ significantly from normal-weight patients. However, underweight patients had consistently the worst outcomes after ischemic stroke.

Several studies reported a lower mortality in obese patients than normal-weight patients after stroke^9–15^. Doehner *et al*. also found obesity predicted a decreased risk of severe disability^15^. While in our study, the risks of death and death or high dependency after ischemic stroke in overweight and obese patients did not differ significantly from that of normal-weight patients. In the surviving patients, overweight and obesity was not associated with better functional recovery, either. The discrepancy of the results may be due to several reasons. First, our study included only subjects after ischemic stroke, whereas some previous studies included also subjects with hemorrhagic stroke^10, 12, 14, 15^. Taking into account the significant differences in pathogenesis and prognostic pattern, combining ischemic and hemorrhagic stroke was not appropriate for the evaluation of the relationship between obesity and ischemic stroke outcome. Second, our analysis controlled for a wide range of covariates, including stroke severity and socioeconomic status, which were the important confounders of the association of obesity and stroke outcome but often absent in previous studies^10–14, 16–18^. In a cohort of Korean patients with ischemic stroke, the favorable effects of overweight and obesity disappeared after adjustment of initial stroke severity^18^. Third, the outcomes were evaluated at different time point. Those studies which found a benefit of obesity often assessed the mortality and functional outcome after a relatively long follow-up period, ranging from 30 months to 10 years^9–12, 15, 26^. Considering that many other diseases might influence the survival and functional status of stroke patients within a longer follow-up period and limited information of these co-morbidities was collected and adjusted in previous analyses^9–12, 15, 16, 26^, their findings were more vulnerable to confounding bias. In contrast, our study selected the mortality within 12 months and the proportion of death or high dependency at 3 and 12 months after stroke as the primary outcomes, which were well-recognized measures of stroke outcome and used as study endpoints in many clinical trials on stroke^27, 28^. So our result was a more reliable estimate of the independent impact of obesity on stroke prognosis. Another study using stroke-related mortality at 1 week and 1 month post-stroke as the outcomes also found no survival advantage in obese patients^16^, which was consistent with our findings. Additionally, the sensitivity analysis with a different definition of high dependency validated our result on functional outcome.

Although we found no significant differences of survival and functional disability in overweight and obese patients compared with normal-weight patients, our results still support the recommendation of current guidelines to strive for normal weight after stroke, given the association between weight loss and improvements in major cardiovascular risk factors, including dyslipidaemia, diabetes and hypertension^19, 29, 30^.

Our study showed underweight patients had the highest all-cause mortality and the worst functional recovery after stroke, which was consistent with previous studies^8, 9, 11, 12, 26^. Although underweight individuals may more often have serious systemic diseases or cancer^16^, our analysis found the distribution of causes of death in underweight group did not differ from that of other BMI subgroups significantly, which indicated the increased mortality associated with underweight could not be attributed to other co-morbidities.

Given the BMI distribution in Asian population is left-shifted as compared with that of Western population^26, 31^, our study utilized the obesity criteria according to the Chinese Guidelines on Prevention and Control of Obesity in Adults^23, 24^. Additionally, the current WHO obesity definition was used in the sensitivity analysis and did not change our results^25^.

Stroke severity is closely associated with the prognosis after stroke^16–18, 32^. Although the National Institutes of Health Stroke Scale (NIHSS) score was not collected in the ChinaQUEST database, we included GCS score and OCSP classification as confounders in our analyses, which had been used as measures of stroke severity in previous studies and had high inter-observer reliability^33–35^. Additionally, GCS score and OCSP classification had been validated as the independent predictors of stroke outcome in the ChinaQUEST cohort^22^.

Some studies indicated smoking was an important confounder of the relation between BMI and mortality^3^. To avoid the potential influence of smoking, we performed a subgroup analysis to assess the association of BMI and stroke outcome in current smokers and current non-smokers, respectively. The analysis showed there was no advantage of survival or functional recovery associated with overweight and obese in both subgroups, which supported our main findings.

There are several limitations to the present study that should be addressed. First, our study is an observational investigation that suffers from the typical limitations of such studies. We adjusted the outcome analyses for a range of identified confounding variables; however, the findings may be influenced by unknown confounders. Second, we could not exclude the possibility of bias due to other treatment and intervention that might influence stroke outcome, although the sensitivity analysis showed that the intravenous thrombolysis treatment did not affect the results of our main analysis. Third, the definition of ‘overweight’ and ‘obesity’ was based on BMI in our study. However, BMI does not reflect body fat distribution, which is also important to the evaluation of obesity^36^. Further studies with measures of body fat distribution, such as waist circumference or waist-to-hip ratio, may provide a more accurate assessment of the association between obesity and stroke outcome.

In conclusion, the present study shows that there is no survival advantage of overweight or obesity after ischemic stroke. The risk of death and functional disability is not significantly different in overweight and obese patients as compared with normal-weight patients. Underweight is associated with the highest risk of death and high dependency after stroke. Our results support the rationality of weight control for overweight and obese patients recommended by current guidelines on secondary prevention of stroke. However, the observational nature of our study warrants further validation.
